# Positive and Negative Symptom Disorder (PND) still the best solution?

**DOI:** 10.1007/s00406-025-02168-9

**Published:** 2025-12-08

**Authors:** Stefan Leucht, Deniz S. Yurtseven, Jim van Os

**Affiliations:** 1 Department of Psychiatry and Psychotherapy, School of Medicine and Health, Technical University of Munich, TUM University Hospital Rechts der Isar, Ismaningerstrasse 22, 81675 Munich, Germany; 2 German Center for Mental Health (DZPG), Partner Site Munich/Augsburg, Munich, Germany; 3 Department of Psychiatry, UMC Utrecht Brain Center, University Medical Center Utrecht, Utrecht University, Utrecht, the Netherlands; 4 Department of Psychosis Studies, Institute of Psychiatry, Psychology and Neuroscience, King’s College London, London, UK

We are grateful for the letters to the editor about our editorial on changing the name for “schizophrenia”, and approximately 80 comments via e-mails. This is exactly the discussion we wanted to stimulate. Nevertheless, no author made a suggestion for an alternative name, and, ost of the points raised by the commentators had already been discussed in our editorial which we recommend to read again [1]. Positive and Negative Symptom Disorder is an attempt to do justice to multiple requirements of a name including brevity.

Hirjiak and colleagues [2] argue that the psychomotor symptoms of schizophrenia should be reflected in the name. However—as the authors themselves note this in another article [3]—the trend is increasingly toward classifying motor symptoms primarily as an independent syndrome of catatonia. This has already been accomplished in ICD-11, and DSM-5 is moving in that direction. Moreover, if the term “motor symptoms” does not appear in the title, it does not mean that these symptoms should no longer be considered defining characteristics of schizophrenia. We suggested a name including motor symptoms in the editorial, but it simply becomes very long. Cognitive symptoms are not part of a name suggestion, although they are even more important—patients with schizophrenia perform 1.5 standard deviations worse than healthy controls. Motor symptoms are less common than negative symptoms, positive symptoms, and cognitive symptoms.

De las Cuevas [4] argues that a name should encompass the full phenomenological and clinical complexity, that the spectrum of core features should account for linguistic and cultural diversity, and that it should anticipate the impact on clinical practice, research, and public understanding. Ideally, this would be the case, but it seems impossible to us. In principle, naming after a person, for example “Bleuler disorder” would solve the problem by not referring to symptoms at all. Such a name would be neutral and it would leave room to any later discoveries on pathophysiology, decisions on defining symptoms etc. However, the WHO no longer wishes to name disorders after individuals. Some Spanish colleagues confirmed that a translation of Positive and Negative Symptom Disorder into Spanish is well possible. Every new name will initially sound unfamiliar and therefore meet cognitive dissonance and resistance. But that is also a matter of habit. Kim Mueser and Susan McGurk pointed out to us in an email, it is better in English to say “Positive and Negative Symptom Disorder” rather than the plural “symptoms disorder,” and we adopt that suggestion.

Pelizza and Pupo [5], like the other letters, raise, the question of whether a name change will eradicate stigma. As we wrote in the editorial, probably not entirely, but it might have some effect in that direction.

Among the many thoughtful points raised by Loch [6] we agree that schizophrenia is so heavily stigmatized that it hinders the early detection of individuals at risk, because no one wants to be associated with the label. We also concur that “Positive and Negative Symptom Disorder” aligns more closely with the dimensional approach pursued by RDoC and HiTOP than does the categorical term “schizophrenia.”

Gama-Marques et al. [7] provide some useful comments for which mistakes should be avoided by a new name. They ultimately argue to keep schizophrenia, among others because changing the name would mean “spreading chaos and confusion” [8]. We are more optimistic, because the name changes made in several Asian countries apparently happened relatively smoothly.

Batalla and Bramness [8] correctly point out that drug-induced psychoses were not considered by us. One reason for not including them was because in the DSM they are dealt with in the chapter “Psychotic Disorders,” whereas in the ICD they are placed outside the psychotic disorders under the respective substances—for example, alcohol-induced psychotic disorder. As Batalla and Bramness, we agree that brief acute and transient psychotic disorders should be captured under brief PND. However, we would prefer to classify substance-induced psychotic disorders as substance-induced PND, i.e., in a separate category.

In principle it is always easier to be against something than to be for it and, correspondingly, none of the commentators has proposed an alternative name. If one wants to cover the current understanding of schizophrenia according to ICD and DSM, one would have to use positive, negative, cognitive, and motor symptom disorder—and such a term appears in our table. We believe that “positive” and “negative” cover the other two domains. Cognitive symptoms are can largely be subsumed under negative symptoms. Motor symptoms can be grouped into too much (hyperkinetic, e.g. agitation — positive) and too little (hypokinetic, e.g. stupor — negative).

We are open to any name, including retaining the term schizophrenia. What is important to us is that the process be systematic, that all relevant aspects are considered, and that the decision be rational. Previous name proposals have not consistently taken all aspects into account. Ultimately, a consensus involving all stake-holders, in particular including people with lived experience is necessary.

## Data Availability

No datasets were generated or analysed during the current study.
